# Comparative data on different preparation methods of Ru/CeO_2_ catalysts for catalytic oxidation of chlorine-containing volatile organic compounds

**DOI:** 10.1016/j.dib.2024.111175

**Published:** 2024-11-26

**Authors:** Pengpeng Cai, Haitao Li, Haibo Zou, Yunkun Pan, Yaoqin Han, Caihe Tang, Yuting Yang, Linghan Xiao, Wei Zheng, Meng Zhou, Jin Li, Mingchun Wu, Daqing Huang, Bing Han

**Affiliations:** a Yueyang Xingchang Petrochemical Co., Ltd., Yueyang 414000, PR China; b Hunan Litai Environmental Engineering Co., Ltd., Chinese Academy of Sciences Eco-Environmental Research Center, Research Laboratory of Advanced Catalysis and Reaction Engineering, Ningxia University, Yueyang 414000, PR China

**Keywords:** Dataset, Chlorinated volatile organic compounds (CVOCs), Sol–gel method

## Abstract

Under industrial conditions, efficient catalytic oxidation of Chlorinated volatile organic compounds is an important challenge, not only because of the poisonous effect of Chlorinated volatile organic compounds on catalysts, but also because of their high reaction temperature, which has an adverse impact on industrialization. In a recent article (*The efficient and stable catalytic combustion of chlorobenzene utilizing a cordierite honeycomb ceramic* Ru/CeO_2_*catalyst: Transitioning from laboratory innovation to practical application*) [1], we developed a strategy for preparing a simple and efficient monolithic catalyst for the catalytic combustion of chlorobenzene. Ru/CeO_2_ was loaded on the industrial support cordierite by a Sol-gel method. Characterization was performed by techniques such as X-ray diffraction (XRD), scanning electron microscopy (SEM), and Brunner-Emmet-Teller(BET) measurements surface area analysis. The Sol-gel method demonstrated superior performance, yielding catalysts with better dispersion, larger surface areas, and consequently, higher catalytic oxidation activity for chlorobenzene, compared to the other two methods. Catalytic tests revealed that the Ru/CeO_2_ catalyst prepared by the Sol-gel method maintained a 99 % conversion rate of chlorobenzene at 500 °C over 80 h, showcasing remarkable stability and resistance to deactivation. This efficacy is attributed to the enhanced dispersion of Ru and the effective interaction between Ru and CeO_2_, facilitated by the Sol-gel synthesis process. This method is simple and easy to prepare the catalyst and has broad industrial prospects. The data set is supplemented with XRD, XPS, SEM and SEM-EDS images of the material, providing useful supplementary data; activity evaluation data for dichloromethane, 1,2-chloroethane and chloromethane were measured.

Specifications Table

**Table:** 

Subject	Environmental Science
Specific subject area	Environmental air pollution and Health
Type of data	Figure, Table, Raw, Analyzed, Filtered, Processed
Data collection	- X-ray diffraction (XRD) was conducted using an X-ray diffractometer (X'Pert Pro MPD) equipped with a Cu Kα source, and the data were collected in the 2θ range of 10–80° diffractograms. - X-ray photoelectron spectroscopy (XPS) was performed using a Thermo Scientific K-Alpha multifunctional surface molecular electron spectrometer from the United States. - The morphology of spent catalysts was examined using a scanning electron microscope (SEM, Zeiss sigma 300). - The effluent gas was analyzed by an online gas chromatograph with an FID detector. - The Activity testing data of the catalyst were evaluated in a continuous flow microreactor composed of a U-shaped quartz tube with an inner diameter of 20 mm and the exhaust gas was analyzed using an online gas chromatograph with an FID detector. Reaction conditions:300–400 °C, GHSV= 15,000 h^-1^, chlorobenzene = 500 ppm.
Data source location	• Organization: Yueyang Xingchang R&D Center • City/Province/Region: Yueyang City, Hunan Province • Country: China
Data accessibility	https://data.mendeley.com/datasets/39z5hgcttr/1
Related research article	10.1016/j.fuel.2024.132605

## 1 Value of the Data

• Researchers can use this dataset to advance the study of the effects of monolithic catalysts on catalytic oxidation of chlorobenzene, methylene chloride, vinyl chloride, and methylene chloride Catalytic oxidation.
• The dataset provides pairs: Ru/CeO_2_ catalysed oxidation of CVOCs. The use of precious metal catalysts for catalytic oxidation of CVOCs is supplemented with some data, and at the same time, rare earth metals and integral supports have a certain reference role in catalytic oxidation of CVOCs.
• This dataset can be used as a research basis for the industrial application of CVOCs catalysts and the future commercial production of integral catalysts under working conditions.
• This dataset provides some basic characterisation of Ru/CeO_2_, such as XRD, XPS, SEM, EDS, etc., which can provide some help for related research.

## 2 Background

Although we have explored the effects of monolithic catalysts prepared by different methods on the activity and stability of chlorobenzene in previous studies, there is still a gap in the evaluation of the catalytic combustion activity of other CVOCs. Therefore, we supplemented the activity evaluation data of dichloromethane, 1,2-chloroethane and chloromethane at the same Cl content. At the same time, we supplemented the XRD spectrum of the monolithic catalyst support cordierite and the complete XPS spectrum of the related Ru/CeO_2_ monolithic catalyst prepared by the Sol-gel method [2].

## 3 Data Description

The data in this article are for a monolithic Ru/CeO_2_ catalyst prepared by the Sol–gel method, which is described in detail in *The efficient and stable catalytic combustion of chlorobenzene utilizing a cordierite honeycomb ceramic* Ru/CeO_2_ *catalyst: Transitioning from laboratory innovation to practical application* [1] and is used for the catalytic combustion of chlorine-containing VOCs.

**Fig. 1:**
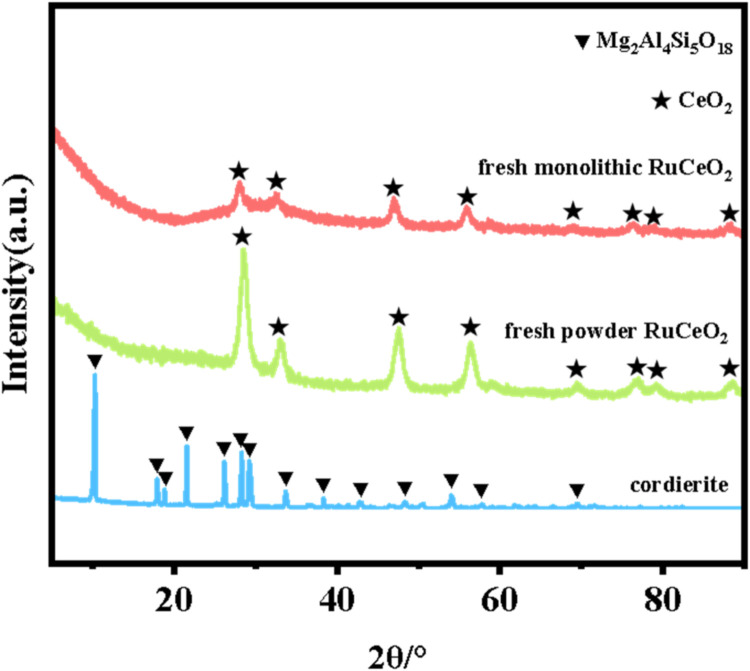
XRD patterns of the Fresh powder and Monolithic Ru/CeO_2_(Sol–gel).

**Fig. 2:**
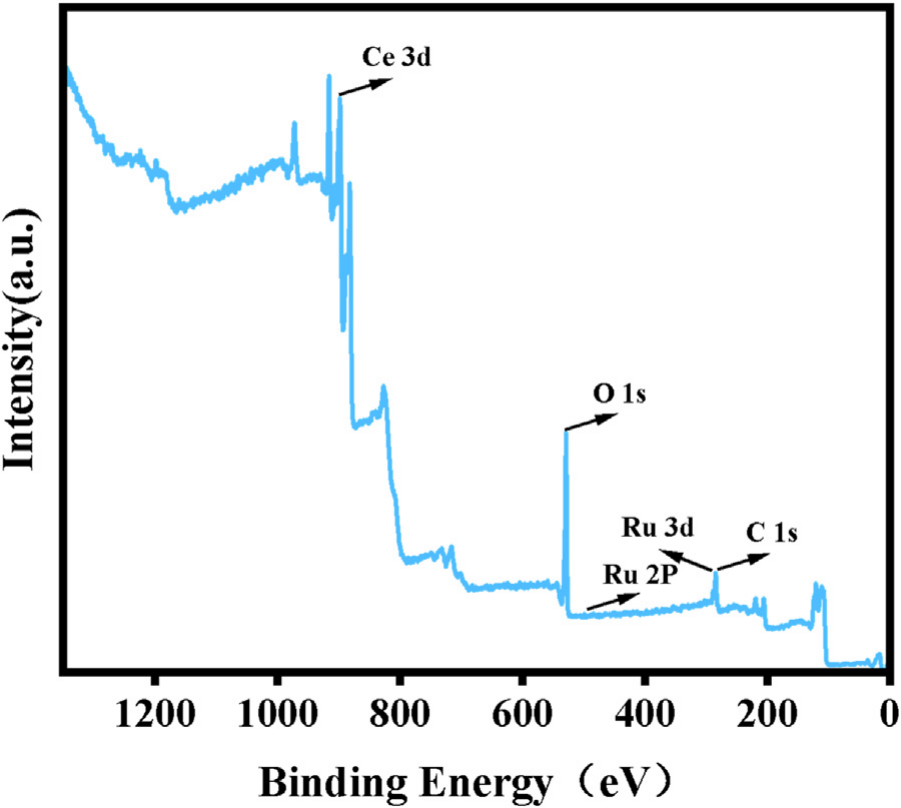
XPS survey spectrum of Ru/CeO_2_(Sol–gel).

**Fig. 3:**
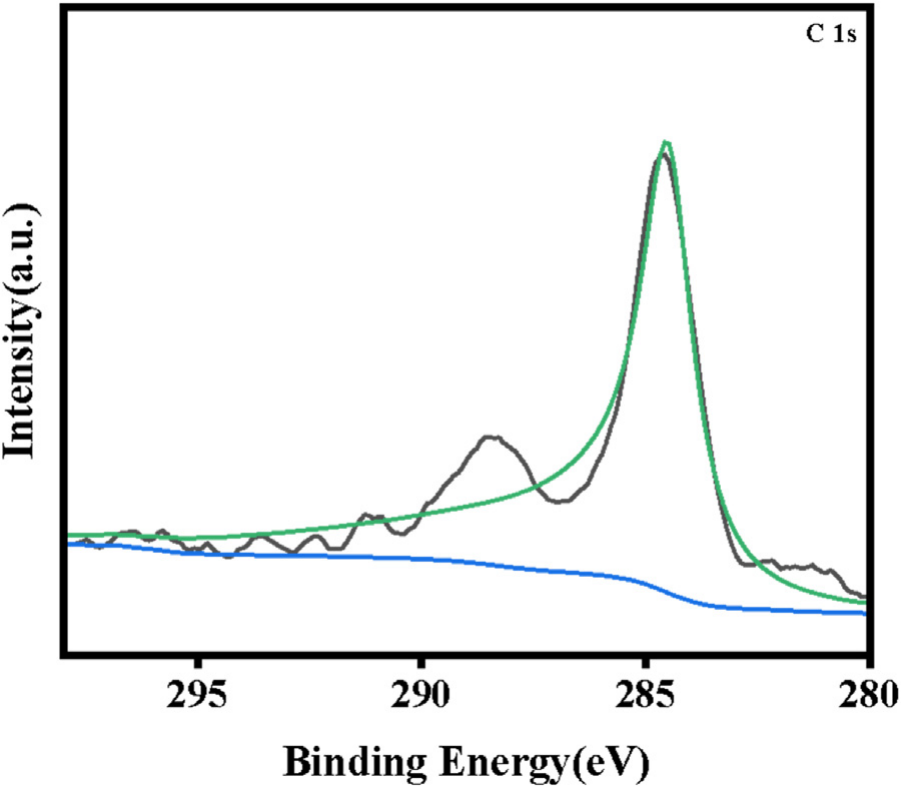
XPS C 1 s spectra of the Ru/CeO_2_(Sol–gel).

**Fig. 4:**
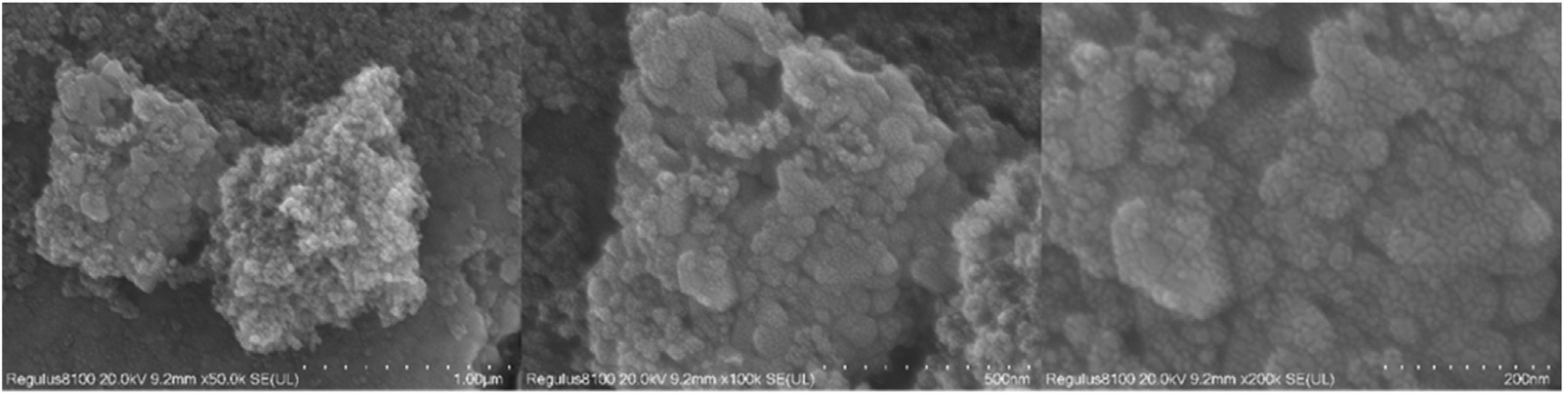
SEM images of the used Ru/CeO_2_(Sol–gel).

**Fig. 5:**
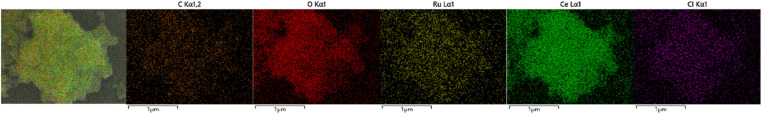
EDS mapping of used Ru/CeO_2_(Sol–gel).

**Fig. 6:**
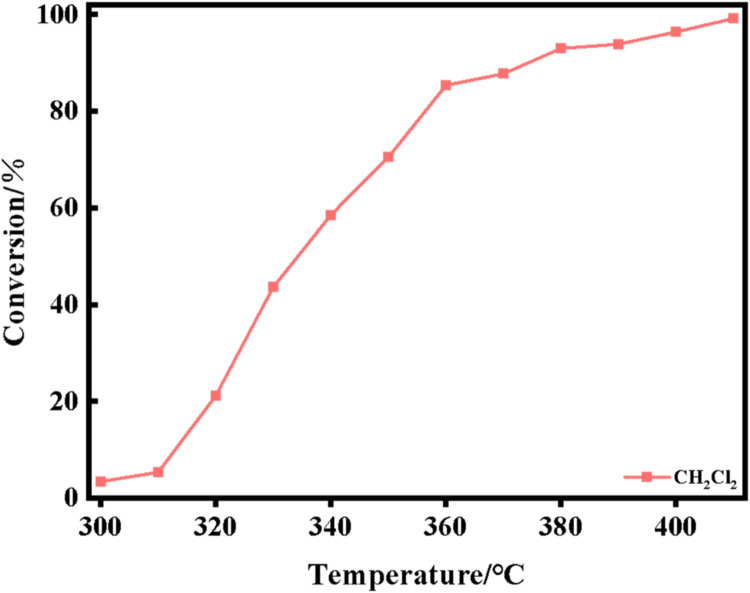
CH_2_Cl_2_ oxidation activity of the Ru/CeO_2_(Sol–gel) catalysts. Reaction conditions: (1) Catalyst loading: 0.2 g; (2) CH_2_Cl_2_=250 ppm; (3) GHSV=15,000 h^-1^; (4) Atmospheric pressure.

**Fig. 7:**
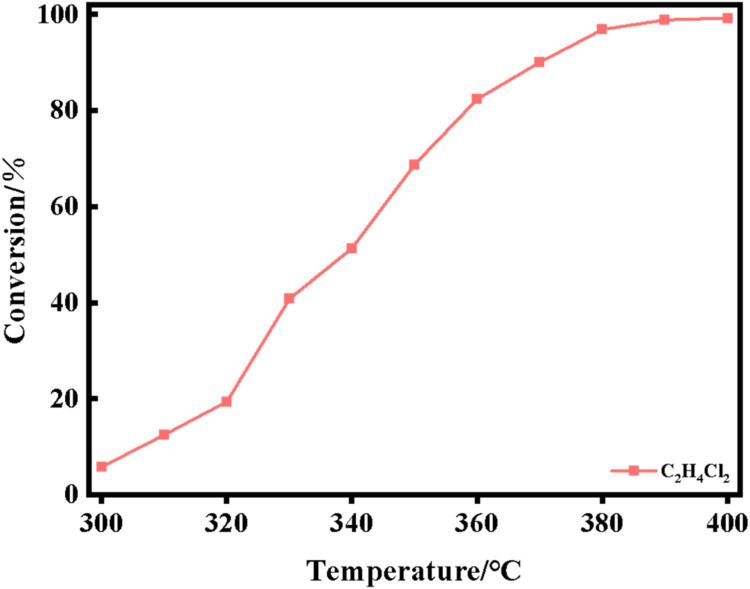
C_2_H_4_Cl_2_ oxidation activity of the Ru/CeO_2_(Sol–gel) catalysts. Reaction conditions: (1) Catalyst loading: 0.2 g; (2) C_2_H_4_Cl_2_=250 ppm; (3) GHSV=15,000 h^-1^; (4) Atmospheric pressure.

**Fig. 8:**
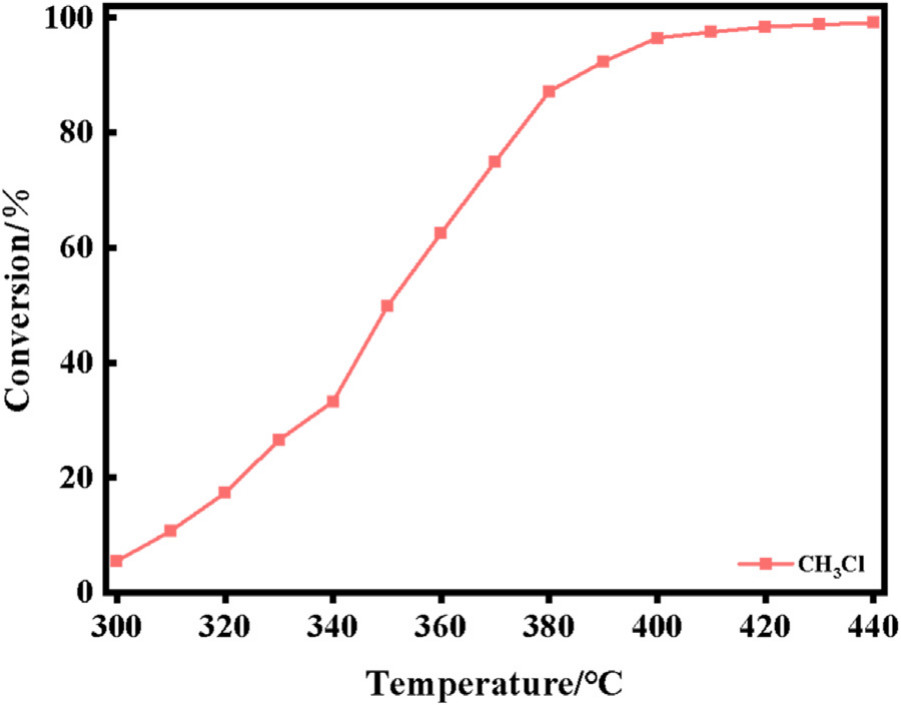
CH_3_Cl oxidation activity of the Ru/CeO_2_(Sol–gel) catalysts. Reaction conditions: (1) Catalyst loading: 0.2 g; (2) CH_3_Cl=500 ppm; (3) GHSV=15,000 h^-1^; (4) Atmospheric pressure.

**Fig. 9:**
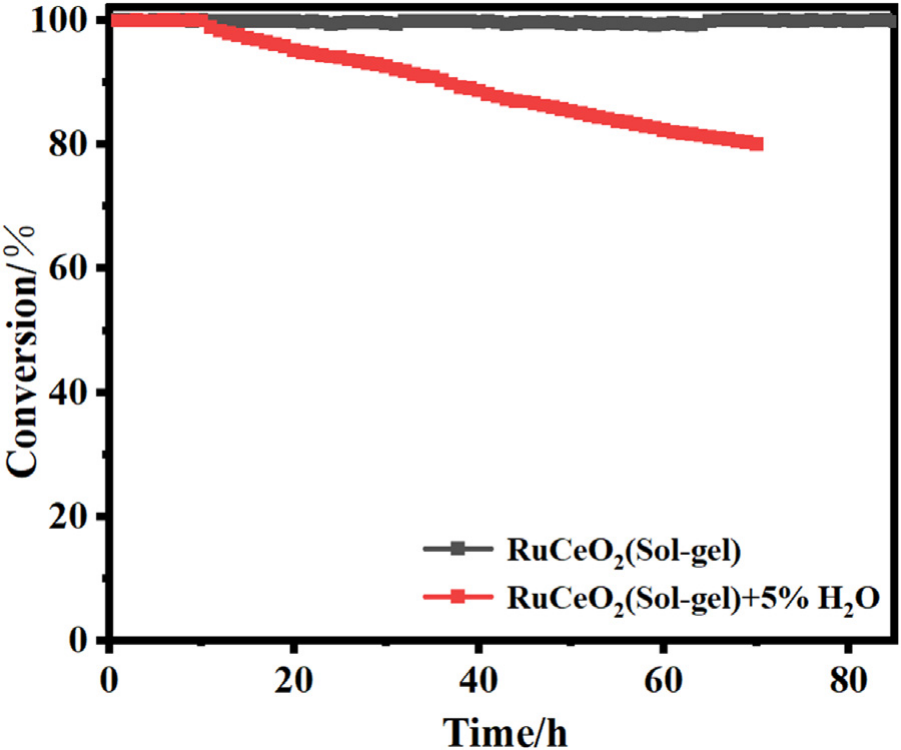
CB stability of the Ru/CeO_2_(Sol-gel) catalysts with 5 vol % water. Reaction conditions: (1) Catalyst loading: 0.2 g; (2) CB=500 ppm; (3) GHSV=15,000 h^-1^; (4) Atmospheric pressure.

The XRD spectra of the powdered Ru/CeO_2_ sample and the monolithic Ru/CeO_2_ sample in Fig. 1 show that the preparation of the monolithic Ru/CeO_2_ catalyst and the powdered Ru/CeO_2_ catalyst have a significant effect on the crystallinity of the catalyst but have no effect on the diffraction peaks of the catalyst sample [3]. The XRD of the support cordierite shows that its main component is Mg_2_Al_4_Si_5_O_18_ [2,4]. The XPS spectrum of the monolithic Ru/CeO_2_ sample (Fig. 2) confirms the presence of the relevant element Ru in Ru/CeO_2_ [5,6]. Fig. 3 supplements the high-resolution overview of the relevant spectral regions (C1s, Ru2p) of the material. Table 1 adds the content of material-related elements. The SEM image in Fig. 4 shows the morphology of the monolithic Ru/CeO_2_(Sol–gel) sample after the reaction [7]. The SEM-EDS image in Fig. 5 shows that Cl appeared in the Ru/CeO_2_(Sol–gel) after the reaction [8]. Fig. 6 shows the activity evaluation of the monolithic Ru/CeO_2_ catalyst prepared by the Sol-gel method at (1) Catalyst loading: 0.2 g; (2) CH_3_Cl=500 ppm; (3) GHSV=15,000 h^-1^; (4) Atmospheric pressure. The activity evaluation of the monolithic Ru/CeO_2_ catalyst prepared by the Sol–gel method is shown in Fig. 7. (1) Catalyst loading: 0.2 g; (2) CH_2_Cl_2_=250 ppm; (3) GHSV=15,000 h^-1^; (4) Atmospheric pressure. The activity evaluation of the monolithic Ru/CeO_2_ catalyst prepared by the Sol–gel method is shown in Fig. 8. (1) Catalyst loading: 0.2 g; (2) C_2_H_4_Cl_2_=250 ppm; (3) GHSV=15,000 h^-1^; (4) Atmospheric pressure. The stability evaluation of the monolithic Ru/CeO_2_ catalyst prepared by the Sol–gel method is shown in Fig. 9. (1) Catalyst loading: 0.2 g; (2) CB=500 ppm; (3) GHSV=15,000 h^-1^; (4) H_2_O=5 vol% (5) Atmospheric pressure. Table 2 summarizes the catalytic activity and stability of Ru-based catalysts reported in the literature for CB catalytic oxidation.

**Table 1:** Total spectrum of distribution plots.

Element	Thread type	Apparent concentration	k Ratio	wt%	wt% Sigma	At%
C	K line	0.98	0.00981	20.84	0.57	39.78
O	K line	7.64	0.02570	34.78	0.35	52.17
Cl	K line	0.13	0.00110	0.39	0.06	0.27
Ru	L line	0.44	0.00435	1.56	0.16	0.38
Ce	L line	13.96	0.12996	42.43	0.37	7.4
Total:				100.00		100.00

**Table 2:** Catalytic oxidation performance of Ru-based catalysts for CB.

Sample	Ru(wt%)	CB(ppm)	T 90(°C)	WHSV (mLg^-1^h^-1^)	Stable(h)	Ref
Ru/CeO_2_/ cordierite	1	500	370	15,000	80	present work
RuCe/SiAlO	0.5	300	278	130,000	190	[9]
Ru/TiO_2_	1	500	287	60,000	10	[10]
Ru/OMS-110	0.8	1000	290	15,000	100	[11]
Ru/Cr_2_O_3_	1	1000	290	120,000	–	[12]
Ru/CeTiO_2_	1	500	279	60,000	10	[13]
Ru/TiO_2_	1	200	275	48,000	16.6	[14]

## 4 Experimental Design, Materials and Methods

The data in this article are for a monolithic Ru/CeO_2_ catalyst prepared by the Sol-gel method, which is described in detail in *The efficient and stable catalytic combustion of chlorobenzene utilizing a cordierite honeycomb ceramic* Ru/CeO_2_ *catalyst: Transitioning from laboratory innovation to practical application* [1] and is used for the catalytic combustion of chlorine-containing VOCs.

### 4.1 X-ray diffraction (XRD)

X-ray diffraction (XRD) was conducted using an X-ray diffractometer (X'Pert Pro MPD) equipped with a Cu Kα source, and the data were collected in the 2θ range of 10–80° diffractograms.

### 4.2 X-ray photoelectron spectroscopy (XPS)

X-ray photoelectron spectroscopy (XPS) was performed using a Thermo Scientific K-Alpha multifunctional surface molecular electron spectrometer from the United States. The Ka (1253.6 eV) of the Mg target was used as the X-ray target source, the vacuum was 10–7 Pa, and the pass energy was 20 eV The C1 s correction peak of contaminated carbon (binding energy is 284.8 eV) was used to correct the XPS spectrum.

### 4.3 Scanning electron microscopy (SEM)

A scanning electron microscope with EDS (SEM, Zeiss sigma 300) was used to examine the surface morphology of the catalyst. A sample of iolite RuCeO2 was taken after use to observe the surface structure changes of the sample after use. Gold is sprayed on the front of the sample to increase sample conductivity for enhanced magnification imaging. SEM analysis is run at 10 kV.

### 4.4 Performance evaluation of catalysts

The catalytic combustion reaction was carried out at atmospheric pressure in a continuous flow microreactor consisting of a U-shaped quartz tube with an inner diameter of 20 mm. A volume of 14.2 ml of catalyst was placed at the bottom of the U-shaped microreactor. The feed flow rate through the reactor was set at 3550 cm^3^ min ^−1^, and the gas hourly space velocity (GHSV) was maintained at 15,000 h ^−1^. The feed flow into the reactor was prepared by delivering liquid dichloromethane, 1,2-dichloroethane into dry air through a syringe pump and metered by a mass flow controller.

The injection point was electrically heated to ensure complete evaporation of the liquid reaction raw materials. The concentration of dichloromethane and 1,2-dichloroethane in the reaction feed was set to 250 ppm. For chloromethane gas, its concentration was set to 500 ppm, and the temperature of the reactor was measured using a thermocouple located at the bottom of the microreactor. The effluent gas was analyzed using an online gas chromatograph with an FID detector. The catalytic activity was measured in the range of 300–500 °C, and the conversion data were calculated based on the difference in inlet and outlet concentrations. After 10 min at each tested temperature, the conversion was measured, and the product curves were obtained.

## Limitation

It is necessary to acknowledge the limitations of this work. Because this study aims to catalyse the oxidation of several CVOCs such as chlorobenzene, dichloromethane, and vinyl chloride in CVOCs, we have limited data, which may affect the generality of this work, and more CVOCs need to be tested and evaluated in the future.

## Ethics Statement

All authors of this article have read and complied with the ethical requirements for publishing in Data in Brief and confirm that the current work does not involve human subjects, animal experiments, or any data collected from social media platforms.

## Credit Author Statement

**Pengpeng Cai:** Writing - Original draft preparation; **Haitao Li:** Investigation, data curation; **Haibo Zhou:** Validation, Supervision; Valerio; **Yaoqing Han:** Visualization; **Yukun Pan:** Data curation; **Caihe Tang:** Validation, Data curation; **Yuting Yang:** Validation; Linghan Xiao; Data curation; **Wei Zheng:** Investigation; **Meng Zhou:** Investigation; **Jin Li:** Data curation; **Mingchun Wu:** Data curation; **Daqing Huang:** Visualization; **Bing Han:** Writing - reviewing & editing.

## Data Availability

Mendeley DataComparative data on different preparation methods of RuCeO2 catalysts for catalytic oxidation of chlorine-containing volatile organic compounds (Original data).

## References

[1] Cai P.P., Li H.T., Han Y.Q., Pan Y.K., Han B. (2024). The efficient and stable catalytic combustion of chlorobenzene utilizing a cordierite honeycomb ceramic RuCeO_2_ catalyst: transitioning from laboratory innovation to practical application. Fuel.

[2] El-Shobaky H.G., Fahmy Y.M. (2006). Nickel cuprate supported on cordierite as an active catalyst for CO oxidation by O_2_. Appl. Catal. B: Environ..

[3] Ding Y., Huang L., Zhang J., Guan A., Wang Q., Qian L., Zhang L., Zheng G. (2020). Ru-doped, oxygen-vacancy-containing CeO_2_ nanorods toward N_2_ electroreduction. J. Mater. Chem. A.

[4] Deng L., Huang C., Kan J., Li B., Chen Y., Zhu S., Shen S. (2018). Effect of coating modification of cordierite carrier on catalytic performance of supported NiMnO_3_ catalysts for VOCs combustion. J. Rare Earths.

[5] Wang Z., Huang Z.P., Brosnahan J.T., Zhang S., Guo Y.L., Guo Y., Wang L., Wang Y.S., Zhan W.C. (2019). Ru/CeO_2_ catalyst with optimized CeO_2_ support morphology and surface facets for propane combustion. Environ. Sci. Technol..

[6] López-Rodríguez S., Davó-Quiñonero A., Bailón-García E., Lozano-Castelló D., Bueno-López A. (2021). Effect of Ru loading on Ru/CeO_2_ catalysts for CO_2_ methanation. Mol. Catal..

[7] Wu J.Y., Chen B., Yan J.R., Zheng X., Wang X.Y., Deng W., Dai Q.G. (2022). Ultra-active Ru supported on CeO_2_ nanosheets for catalytic combustion of propane: experimental insights into interfacial active sites. Chem. Eng. J..

[8] Lin B.Y., Liu Y., Heng L., Wang X.Y., Ni J., Lin J.X., Jiang L.L. (2018). Morphology effect of ceria on the catalytic performances of Ru/CeO_2_ catalysts for ammonia synthesis. Ind. Eng. Chem. Res..

[9] Yu C., Gu J.-n., Xue Y., Guo M., Li K., Jia J., Sun T. (2024). Hierarchical molecular sieve-based Ce-Ru oxide for enhanced catalytic oxidation of chlorobenzene: insight into the synergistic effect of Ce and Ru, the role of molecular sieve. Process Saf. Environ. Prot..

[10] Liu X., Chen L., Zhu T., Ning R. (2019). Catalytic oxidation of chlorobenzene over noble metals (Pd, Pt, Ru, Rh) and the distributions of polychlorinated by-products. J. Hazard Mater..

[11] Yang S., Zhao H., Dong F., Tang Z., Zha F. (2019). Highly efficient catalytic combustion of o-dichlorobenzene over lattice-distorted Ru/OMS-2: the rapidly replenishing effect of surface adsorbed oxygen on lattice oxygen. Mol. Catal..

[12] Chen X., Jia Z., Liu Z., Wang X., Liang M. (2023). Strong metal-support interactions between atomically dispersed Ru and CrO for improved durability of chlorobenzene oxidation. RSC Adv..

[13] Ye M., Chen L., Liu X., Xu W., Zhu T., Chen G. (2018). Catalytic oxidation of chlorobenzene over ruthenium-ceria bimetallic catalysts. Catalysts.

[14] Sun B., Li Q., Su G., Song M., Ma C., Pang J., Zhao X., Meng J., Shi B. (2025). Sustainable and efficient catalytic oxidation of chlorinated volatile organic compounds over Ru-loaded facet-engineered {201}-TiO_2_ catalyst with tuned defects. Appl. Catal. B: Environ. Energy.

